# Social Isolation, Genetic Susceptibility, Systemic Inflammation and Risk of Abdominal Aortic Aneurysm: A UK Biobank Cohort Study

**DOI:** 10.3390/healthcare14142126

**Published:** 2026-07-15

**Authors:** Yu Xia, Xinyi Liu, Yongliang Zhong, Zhiyu Qiao, Haiou Hu, Chengnan Li, Yipeng Ge, Junming Zhu

**Affiliations:** Department of Cardiovascular Surgery, Beijing Aortic Disease Center, Beijing Anzhen Hospital of Capital Medical University, Beijing 100029, Chinaliuxinyi@mail.ccmu.edu.cn (X.L.); zhongyl2012@yeah.net (Y.Z.); yipengge_anzhen@126.com (Y.G.)

**Keywords:** abdominal aortic aneurysm, social isolation, polygenic risk score, systemic inflammation response index, cohort study

## Abstract

Background: Abdominal aortic aneurysm (AAA) is typically asymptomatic until rupture, and established risk factors do not fully explain its occurrence. Whether social isolation, loneliness, polygenic susceptibility, and systemic inflammation jointly influence AAA risk remains unclear. Methods: We included 356,181 White British participants from the UK Biobank free of AAA at baseline. Social isolation and loneliness indices were derived from questionnaires. A genome-wide polygenic risk score (PRS) for AAA was constructed using PRS-CS and grouped into tertiles. The systemic immune-inflammation index (SII) and systemic inflammation response index (SIRI) were calculated from baseline blood counts. Incident AAA was identified through linked hospital, primary care, mortality, and self-report records. Cox models estimated hazard ratios (HRs), and mediation analyses quantified the proportion of the social isolation–AAA association explained by inflammatory indices. Results: Over 15.6 years of follow-up, 1727 AAA events occurred. A social isolation index ≥2 was associated with higher AAA risk (HR 1.23; 95% CI 1.05–1.46), and each 1-SD increase corresponded to ~11% higher risk; loneliness was not associated with AAA. Although statistical interactions were non-significant, individuals with both high PRS and high social isolation had fourfold higher risk than those with low PRS and no isolation. SIRI, but not SII, was independently associated with AAA (HR 1.24; 95% CI 1.06–1.46) and mediated ~5% of the association. Conclusions: Social isolation, particularly in genetically susceptible individuals, identifies a population at substantially elevated AAA risk, partly via systemic immune–inflammatory activation, and may represent a modifiable target for prevention.

## 1. Introduction

Abdominal aortic aneurysm (AAA) is a chronic degenerative disorder of the abdominal aorta, defined by a focal enlargement surpassing 3 cm in diameter or 50% above its typical size [1]. Because AAA often remains asymptomatic until rupture, contemporary guidelines emphasize ultrasound-based surveillance, cardiovascular risk reduction, and timely elective repair according to aneurysm diameter, growth rate, symptoms, and individualized operative risk. Nevertheless, rupture remains life-threatening, highlighting the importance of identifying high-risk individuals before clinical presentation [2,3]. Although age, male sex, cigarette smoking, hypertension, and dyslipidemia are well-recognized determinants, these factors do not entirely explain AAA development or its progression [4]. Consequently, there is a pressing need to identify additional genetic and modifiable influences to enhance risk prediction and inform preventive measures.

Social isolation and loneliness, two related but distinct constructs, have been increasingly recognized as psychosocial determinants of cardiovascular health. Meta-analyses show that poor social connectedness is associated with higher risks of coronary heart disease, stroke, and mortality [5]. Biological studies suggest that social disconnection may promote adverse neuroendocrine activation, heightened sympathetic tone, systemic inflammation, endothelial dysfunction, and unhealthy behaviors [6]. These pathways are relevant to AAA because aneurysm formation is characterized by chronic vascular inflammation, macrophage and myeloid-cell infiltration, extracellular matrix degradation, and vascular smooth muscle cell dysfunction [7,8]. Recent UK Biobank proteomic evidence further suggests that social isolation and loneliness are associated with circulating proteins involved in inflammation, immune activation, and complement pathways [9]. However, direct prospective evidence linking social isolation or loneliness to AAA remains extremely limited. Therefore, whether social disconnection contributes to AAA development through behavioral and immune-inflammatory pathways remains unclear.

AAA has a substantial genetic basis, with large genome-wide association studies identifying multiple risk loci related to extracellular matrix remodeling, lipid metabolism, and vascular inflammation [10]. Polygenic risk scores (PRS) derived from these variants can effectively predict AAA risk beyond traditional risk factors [11]. However, it remains unclear whether genetic susceptibility amplifies the adverse effects of psychosocial factors such as social isolation or loneliness. Systemic and local vascular inflammation play central roles in AAA pathophysiology, characterized by neutrophil and monocyte/macrophage recruitment, protease-mediated extracellular matrix degradation, oxidative stress, and vascular smooth muscle cell dysfunction [12]. Composite inflammatory indices such as systemic immune-inflammation index (SII) and the systemic inflammation response index (SIRI), which integrate multiple blood cell counts, have emerged as important indicators of low-grade chronic inflammation and cardiovascular risk [13]. Nevertheless, whether social factors, genetic susceptibility, and systemic inflammation interact or mediate each other, and how these pathways collectively influence AAA risk, has not yet been systematically investigated.

This study aims to examine the associations between social isolation, loneliness, and the risk of abdominal aortic aneurysm in the UK Biobank prospective cohort. In addition, it investigates whether social factors interact with polygenic susceptibility to modify AAA risk, and evaluates the extent to which systemic inflammatory markers, including the SII and the SIRI, mediate these relationships. By integrating behavioral, psychosocial, inflammatory, and genetic dimensions, this research seeks to clarify the multifactorial determinants of AAA and to identify high-risk groups who may benefit from targeted preventive strategies.

## 2. Materials and Methods

### 2.1. Study Design and Participants

This study utilized data from the UK Biobank, a nationwide prospective cohort that includes over half a million adults. Between 2006 and 2010, individuals aged 37–73 years were recruited from 22 assessment centres throughout the United Kingdom. At enrolment, participants completed an extensive touchscreen questionnaire capturing demographic characteristics, socioeconomic indicators, lifestyle factors, and medical history. Detailed descriptions of the cohort design and data collection procedures have been documented in prior publications [14]. Ethical approval for the UK Biobank was granted by the North West Multi-centre Research Ethics Committee (Ref: 11/NW/0382). All participants signed an electronic consent form at recruitment.

The cohort initially included 466,270 participants, each with complete baseline data on social isolation and loneliness. Participants were excluded if they declined follow-up, lacked information on demographic, socioeconomic, lifestyle, or inflammatory markers, or had any recorded diagnosis of AAA before the baseline assessment. Baseline AAA was identified using the same diagnostic codes and linked health-care/self-report records as those used for incident outcome ascertainment. Individuals who were not of White British ancestry (defined using self-reported ethnicity and principal component analysis provided by the UK Biobank) were also excluded. The final analytic sample comprised 356,181 participants. A detailed flowchart is presented in Supplementary Figure S1.

### 2.2. Assessment of Social Isolation and Loneliness

Social isolation and loneliness were assessed using self-administered touchscreen questionnaires completed at the UK Biobank assessment center. Following previous UK Biobank studies [15], the social isolation index was constructed from three structural social-connection items: number in household (Field 709), frequency of friend/family visits (Field 1031), and leisure/social activities (Field 6160). Participants were assigned one point for each of the following: living alone, visiting friends or family less than once per month, or not participating weekly in any social or group-based activities, including sports clubs, social clubs, religious groups, or adult education classes. All other responses were coded as 0. Because few participants scored 3 on the isolation scale, scores of 2 and 3 were combined into a single “≥2” category.

Loneliness was assessed using two subjective social-emotional items: often feeling lonely (Field 2020) and frequency of being able to confide in someone close (Field 2110). Participants were assigned one point if they reported often feeling lonely or being able to confide only every few months or less; all other responses were coded as 0. The social isolation score was calculated by summing the three structural items and categorized as 0, 1, or ≥2, whereas the loneliness score was calculated by summing the two subjective items and categorized as 0, 1, or 2. Social isolation and loneliness were analyzed as separate exposures rather than combined into a single psychosocial score.

### 2.3. Polygenic Risk Score Calculation

The polygenic risk score (PRS) for AAA was constructed using PRS-CS, a Bayesian regression framework that estimates posterior SNP effect sizes using continuous shrinkage priors and external linkage disequilibrium (LD) information [16]. Summary statistics were obtained from a GWAS of AAA conducted in individuals of European ancestry, as listed in the GWAS Catalog (GCST90475994), comprising 7642 AAA cases and 172,172 controls. Prior to PRS construction, variants with a minor allele frequency < 0.01 or imputation INFO score < 0.8 were excluded. PRS-CS uses LD information from the 1000 Genomes Project Phase 3 European reference panel to account for polygenicity and correlation among variants. The method was implemented in auto mode, allowing the global shrinkage parameter to be estimated from the data. Individual PRS values were calculated by summing the posterior SNP effect sizes across included autosomal variants for each participant. Based on the distribution of PRS values in the analytic sample, participants were categorized into three strata of genetic susceptibility: low, intermediate, and high, corresponding to the first, second, and third tertiles, respectively. There was no sample overlap between the GWAS discovery cohort and the present UK Biobank participants.

### 2.4. Covariate Assessment

Demographics consisted of age, sex, education, Townsend Deprivation Index (TDI), employment status, and body mass index (BMI). Medical history included medication use; chronic respiratory, liver, and kidney diseases; other cardiovascular diseases; psychiatric diseases; hypertension; diabetes; and dyslipidemia. Lifestyle factors included smoking, alcohol consumption, physical activity, sedentary time, sleep patterns, and diet. Detailed definitions for all lifestyle factors are provided in Supplementary Tables S1 and S2. Medication history was modeled as the number of regular medications reported at baseline, and specific medication classes, including antihypertensive, lipid-lowering, and antidiabetic medication use, were additionally defined according to UK Biobank medication codes, as shown in Supplementary Table S3.

In the UK Biobank, peripheral blood cell counts were measured using a clinically validated Coulter LH 750 analyzer with manufacturer-recommended quality control procedures [17]. Baseline counts of neutrophils, monocytes, platelets, and lymphocytes were obtained. Two composite indices reflecting immune-inflammatory status were calculated: the Systemic Inflammation Response Index (SIRI = neutrophils × monocytes/lymphocytes) and the Systemic Immune-Inflammation Index (SII = neutrophils × platelets/lymphocytes) [18,19,20].

### 2.5. Outcomes

The primary endpoint was incident AAA, identified through ICD-10 codes I71.3 and I71.4 using data from death registries, primary care records, hospital admissions, and participant self-reports. Newly diagnosed cases were defined by the first recorded occurrence of an AAA diagnosis. Follow-up time was measured from baseline assessment until the earliest of the following events: AAA diagnosis, death, loss to follow-up, or the end of the observation period.

### 2.6. Statistical Analysis

Baseline characteristics were summarized as means (SD) for continuous variables and counts (percentages) for categorical variables. Group differences were compared using one-way ANOVA for continuous variables and chi-square tests for categorical variables. Kaplan–Meier curves were generated to display cumulative incidence of AAA across categories of the social isolation and loneliness indices. Cox proportional hazards models were used to estimate hazard ratios (HRs) and 95% confidence intervals (CIs); the proportional hazards assumption was evaluated using Schoenfeld residuals. Three progressively adjusted models were constructed: Model 1: Adjusted for sex and age. Model 2: Further adjusted for education level, employment status, Townsend deprivation index, body mass index, medication history, and medical history (including hypertension, diabetes, dyslipidemia, cardiovascular diseases, psychiatric disease, chronic respiratory diseases, chronic kidney disease, and chronic liver disease). Model 3: Additionally adjusted for smoking status, alcohol consumption, physical activity, diet, sleep patterns, and sedentary time.

Stratified analyses were performed by age, sex, BMI category (18.5–24.9 vs. outside this range), history of hypertension, and smoking status. Multiple sensitivity analyses were conducted to assess the robustness of the findings. First, to minimize potential reverse causality, cases diagnosed within the first two years of follow-up were excluded. Second, to evaluate the influence of baseline comorbidities, we excluded participants with major baseline conditions, including diabetes, hypertension, dyslipidemia, cardiovascular diseases, psychiatric diseases, chronic respiratory diseases, and chronic kidney or liver disease. Third, Fine-Gray subdistribution hazard models were applied to account for competing risks from non-AAA deaths or loss to follow-up. Fourth, multiple imputation by chained equations was used to address missing data in demographic, socioeconomic, and lifestyle covariates. Fifth, considering the potential influence of medication classes related to AAA risk factors, we additionally adjusted for antihypertensive, lipid-lowering, and antidiabetic medication use.

To assess the potential impact of genetic susceptibility, we first examined AAA risk across PRS categories using Cox proportional hazards models. In subsequent analyses involving PRS, all models were additionally adjusted for the first ten genetic principal components provided by the UK Biobank to account for potential residual population stratification and fine-scale ancestry differences, even within participants of White British ancestry. We further evaluated interactions and joint effects of the social isolation index and PRS on AAA risk. Multiplicative interaction was assessed by including a cross-product term between the social isolation index and PRS in the Cox model and reporting HRs with 95% CIs. Additive interaction was evaluated using the relative excess risk due to interaction (RERI) and its 95% CI. In joint effect analyses, the reference group consisted of individuals with low genetic risk and low social isolation. Participants were then stratified by PRS category, and the cumulative incidence of AAA was estimated across categories of the social isolation index within each PRS stratum. Ten-year absolute risk increase (ARI) for AAA was calculated by comparing social isolation groups, and trends in ARI across PRS categories were tested using weighted least-squares models. Finally, causal mediation analysis was performed to evaluate whether systemic inflammatory indices mediated the association between social isolation and AAA risk, adjusting for all covariates included in Model 3. This analysis decomposed the total association into the average causal mediation effect (ACME), representing the indirect effect through the inflammatory index, and the average direct effect (ADE), representing the remaining effect not explained by the inflammatory index. The proportion mediated was calculated as the ratio of ACME to the total effect. To estimate 95% CIs for mediation proportions, we applied 1000 quasi-Bayesian Monte Carlo simulations with bootstrap resampling.

All statistical analyses were conducted using R software (version 4.4.1). Two-sided tests were used, and *p*-values < 0.05 were considered statistically significant.

## 3. Results

### 3.1. Baseline Characteristics of Participants

Baseline characteristics of participants across categories of the social isolation index are shown in Table 1. A total of 356,181 individuals were included in the analysis, with a mean age of 56.6 years (SD = 8.1), and 46.2% were men. Compared with participants with a social isolation index of 0, those with an index ≥ 2 were more likely to be male, have lower educational attainment, exhibit less healthy lifestyle behaviors, have a higher burden of comorbidities, and display elevated inflammatory levels. Supplementary Table S4 presents baseline characteristics stratified by loneliness index, demonstrating a pattern of participant characteristics similar to that observed for the social isolation index.

### 3.2. Associations of Social Isolation and Loneliness with the Risk of Incident AAA

**Figure 1:**
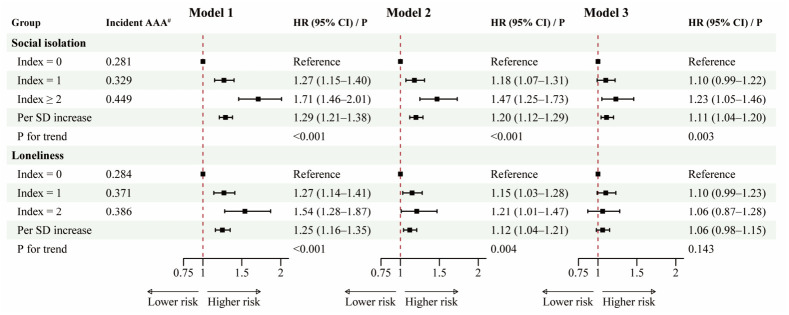
Associations of social isolation and loneliness with the risk of incident AAA. # Incidence rates per 1000 person-years. Model 1: Adjusted for sex and age. Model 2: Further adjusted for education level, employment status, Townsend deprivation index, body mass index, medication history, and medical history (including hypertension, diabetes, dyslipidemia, cardiovascular diseases, psychiatric diseases, chronic respiratory diseases, chronic kidney disease, and chronic liver disease). Model 3: Additionally adjusted for smoking status, alcohol consumption, physical activity, diet, sleep patterns, and sedentary time.

During a mean follow-up of 15.6 years, a total of 1727 incident AAA cases were identified. As shown in the Kaplan–Meier curves in Supplementary Figure S2, higher social isolation and loneliness indices were significantly associated with increased cumulative incidence of AAA. In multivariable Cox regression analyses (Figure 1), participants with a social isolation index ≥ 2 had a higher risk of developing AAA compared with those with an index of 0 (HR = 1.23; 95% CI: 1.05–1.46) in the fully adjusted model. Additionally, each 1-SD increase in the social isolation index was associated with approximately an 11% higher AAA risk. In contrast, the loneliness index showed no significant association with AAA incidence. The proportional hazards assumption was verified using Schoenfeld residuals, with all *p*-values > 0.05 (Supplementary Table S5), supporting the validity and robustness of the analyses. The C-indices ranged from 0.836 to 0.881, with Model 3 showing the best discrimination (highest C-index and lowest Bayesian information criterion).

Subgroup analyses (Supplementary Table S6) indicated no significant interactions between social isolation or loneliness and sex, age, BMI, hypertension status, or smoking status. Multiple sensitivity analyses were conducted to test the robustness of the findings (Supplementary Table S7), including exclusion of cases occurring within the first two years of follow-up, application of competing risk models, exclusion of participants with major baseline comorbidities, multiple imputation of missing data and additional adjustment for antihypertensive, lipid-lowering, and antidiabetic medication use. All results were consistent with the primary findings, further supporting their reliability.

### 3.3. Genetic Risk, Social Isolation, and the Risk of Incident AAA

As shown in Supplementary Figure S3, the AAA polygenic risk score was approximately normally distributed. Based on the tertile cut-off values of −0.048 and −0.036, participants were classified into low, intermediate, and high genetic susceptibility groups. As shown in Supplementary Table S8, participants in the highest PRS tertile had a substantially higher risk of developing AAA compared with those in the lowest tertile (HR = 3.75; 95% CI: 3.28–4.29). Based on the observed strong association between social isolation and AAA incidence, subsequent analyses focused primarily on social isolation.

**Figure 2:**
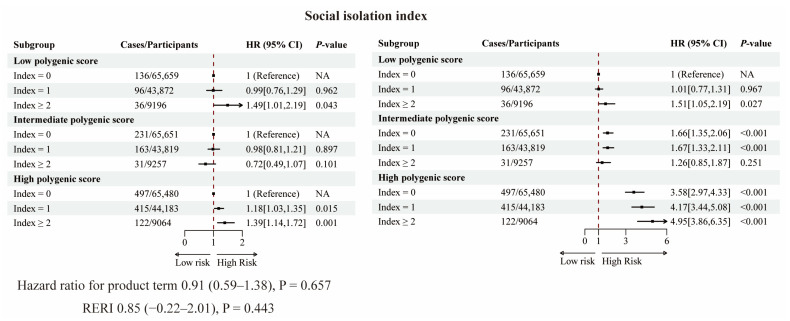
Interaction and joint effects of social isolation and polygenic risk score on the risk of incident AAA. All analyses were adjusted for sex, age, first ten genetic principal components, education level, employment status, Townsend deprivation index, body mass index, medication history, medical history (including hypertension, diabetes, dyslipidemia, cardiovascular diseases, psychiatric diseases, chronic respiratory diseases, chronic kidney disease, and chronic liver disease), as well as smoking status, alcohol consumption, physical activity, diet, sleep patterns, and sedentary time.

**Figure 3:**
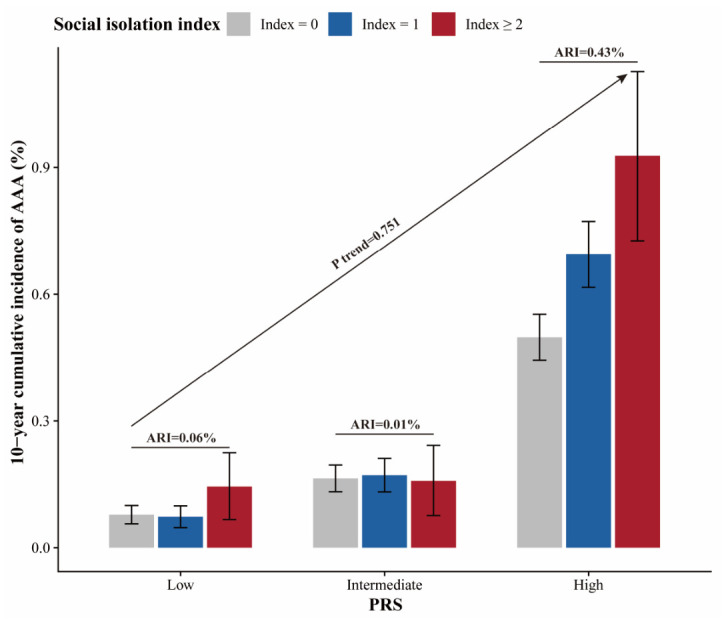
Ten-year cumulative incidence of AAA according to categories of the polygenic risk score and social isolation index. Abbreviations: ARI, absolute risk increase; PRS, polygenic risk score.

We further examined the potential interaction between genetic risk and the social isolation index in relation to AAA incidence. As illustrated in Figure 2, no significant multiplicative or additive interactions were observed between PRS and the social isolation index (all *p*-values > 0.05). However, their joint effects were notable. Specifically, compared with participants in the low-PRS group with a social isolation index of 0, those with high PRS and a social isolation index ≥ 2 had approximately a fourfold higher risk of AAA (HR = 4.95; 95% CI: 3.86–6.35). Additionally, we evaluated the joint associations of PRS and the social isolation index with the 10-year cumulative incidence of AAA (Figure 3). Among individuals in the high-PRS stratum, participants with a social isolation index ≥ 2 had a significantly higher incidence than those with an index of 0, corresponding to an absolute risk increase (ARI) of 0.43%. This pattern was not prominent among participants in the low or intermediate PRS strata. No significant linear trend across PRS categories was observed (trend *p* = 0.751).

### 3.4. Inflammatory Markers, Social Isolation, and AAA Risk

**Figure 4:**
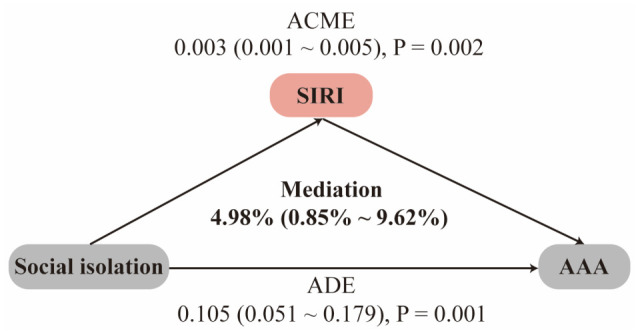
Mediation analysis of the inflammatory index SIRI in the association between social isolation and AAA risk. All analyses were adjusted for sex, age, education level, employment status, Townsend deprivation index, body mass index, medication history, medical history (including hypertension, diabetes, dyslipidemia, cardiovascular diseases, psychiatric diseases, chronic respiratory diseases, chronic kidney disease, and chronic liver disease), as well as smoking status, alcohol consumption, physical activity, diet, sleep patterns, and sedentary time. Abbreviations: ADE, Average direct effects; ACME, Average causal mediation effects.

As shown in Supplementary Table S9, after adjustment for all potential confounders, higher SIRI levels were significantly associated with an increased risk of AAA (HR = 1.24; 95% CI: 1.06–1.46). In contrast, no significant association was observed between SII and AAA risk. We further explored the potential mediating role of SIRI in the association between the social isolation index and AAA risk. As illustrated in Figure 4, SIRI mediated 4.98% of the association between social isolation and AAA risk (95% CI: 0.85–9.62%).

## 4. Discussion

This study found that a higher social isolation index was significantly associated with an increased risk of AAA, whereas loneliness showed no significant association. Although no statistically significant multiplicative or additive interaction between social isolation and PRS was detected, individuals with both high PRS and high social isolation had the greatest risk of AAA in the joint analysis, suggesting a cumulative risk pattern rather than statistical interaction. Notably, among those with high genetic susceptibility, social isolation was particularly associated with an elevated absolute risk of AAA. Importantly, part of the association between social isolation and AAA risk was mediated by higher SIRI levels.

In this large prospective cohort, social isolation, but not loneliness, was significantly associated with a higher risk of incident AAA. This distinction echoes findings in broader cardiovascular research, where social isolation often shows stronger and more consistent associations with adverse cardiometabolic outcomes than subjective loneliness alone [21]. For instance, social isolation has been linked to elevated risks of coronary heart disease, stroke, and all-cause mortality independent of emotional loneliness [22,23]. Several mechanisms may explain this divergence. Structural disconnection typically reflects reduced social resources, limited access to health information, fewer opportunities for physical activity, and lower adherence to medical therapy—all of which have been implicated in AAA risk factors such as smoking persistence, hypertension, and delayed disease detection [24]. Loneliness, in contrast, may exert more psychological than behavioral effects and has shown inconsistent associations with objective cardiovascular diseases [21]. The present study’s findings thus support the growing recognition that objective and subjective social disconnection represent distinct constructs with different biological and behavioral consequences [25]. Given AAA’s long asymptomatic course and dependence on modifiable risk profiles, structural isolation may be especially relevant in influencing long-term aneurysm development.

Although no statistically significant multiplicative or additive interactions between social isolation and PRS were detected, individuals with both high genetic susceptibility and high social isolation exhibited the greatest absolute and relative risk of AAA. This pattern should be interpreted as the co-occurrence of two independent risk dimensions rather than evidence that social isolation modifies genetic susceptibility. From a risk-stratification perspective, this finding suggests that considering psychosocial factors alongside inherited susceptibility may help identify individuals with a higher overall risk burden. Genome-wide association studies have consistently demonstrated the strong polygenic architecture of AAA, identifying loci involved in extracellular matrix biology, lipid metabolism, vascular remodeling, and inflammation [11]. Social isolation may further compound genetic predisposition by amplifying behavioral and inflammatory exposures that converge on these pathways. For instance, individuals experiencing structural isolation often exhibit higher smoking prevalence, physical inactivity, and poorer diet quality [26], factors known to interact with AAA risk loci and increase aneurysmal degeneration. The observed absolute risk increase in the high-PRS group suggests that social isolation may function as a contextual amplifier of inherited vascular vulnerability [27], highlighting an at-risk subgroup that may especially benefit from targeted preventive strategies. However, because the PRS was derived and applied within participants of White British ancestry, these findings should not be directly extrapolated to other ancestral groups. Future studies in more diverse populations are needed, as both the genetic architecture of AAA and the social meaning of isolation may differ across ethnic, cultural, and healthcare contexts.

In addition to the behavioral profile, systemic inflammation partially mediated the association between social isolation and AAA. SIRI, but not SII, was independently associated with AAA risk and accounted for approximately 5% of the effect. These findings are consistent with prior studies showing that composite inflammatory indices integrating neutrophils and monocytes capture low-grade immune activation more sensitively than platelet-based indices [28]. Neutrophil and monocyte infiltration is a hallmark of AAA pathogenesis and contributes to matrix degradation, smooth muscle cell apoptosis, and oxidative stress [29]. Psychosocial stressors such as social disconnection have been shown to upregulate conserved transcriptional responses to adversity, characterized by increased pro-inflammatory gene expression and heightened myeloid cell activity [30]. Although neuroendocrine stress pathways are biologically plausible, stress-related hormonal mediators, such as cortisol or catecholamines, were not evaluated because standardized measurements were not available for the full baseline analytic cohort in UK Biobank [17]. The present mediation results suggest that systemic immune-inflammatory activation serves as one biological pathway through which chronic structural isolation may promote aneurysmal remodeling. Nevertheless, the modest magnitude of mediation indicates that SIRI captures only one component of the social isolation–AAA pathway, and that additional behavioral, neuroendocrine, vascular, and local aortic-wall mechanisms likely contribute to the remaining association.

Several plausible biological and behavioral mechanisms may underlie the observed association between social isolation and AAA. First, chronic social disconnection has been linked to dysregulated hypothalamic–pituitary–adrenal (HPA) axis activity and elevated sympathetic tone, which may contribute to hemodynamic stress, endothelial dysfunction, and vascular stiffness, processes that are biologically relevant to aneurysm formation. [31,32]. Second, structural isolation is associated with lifestyle patterns such as persistent smoking, poor diet quality, and reduced physical activity, all of which are established AAA risk factors [26]. Given the well-established association between smoking and AAA development, although smoking status was adjusted for in our analyses, this categorical measure may not fully capture cumulative smoking exposure, such as pack-years or smoking cessation timing. Third, social isolation is known to activate conserved inflammatory transcriptomic programs, increasing IL-6-, TNF-α-, and NF-κB-related signaling, which may promote macrophage recruitment, MMP-mediated extracellular matrix degradation, and vascular smooth muscle cell dysfunction or apoptosis in the aneurysmal wall [30,33]. These converging pathways may explain why structural isolation, but not loneliness, exhibited a robust association with AAA in this cohort.

This study benefits from a large, well-phenotyped cohort with long-term follow-up, enabling robust assessment of AAA risk. By integrating psychosocial exposures, a genome-wide PRS, and composite inflammatory markers, it provides a comprehensive evaluation of behavioral, genetic, and inflammatory pathways. It is also the first prospective study to link social isolation with incident AAA and to identify a high-risk subgroup defined by both high PRS and high social isolation. However, several limitations merit consideration. AAA diameter information was unavailable, preventing size-based analyses. Residual confounding cannot be fully excluded despite extensive adjustment. In addition, our study was primarily observational and association-based; therefore, further experimental and mechanistic studies are needed to clarify the potential pathways underlying these associations. Social isolation and loneliness were assessed by questionnaires, which may not reflect long-term patterns. Finally, the restriction to White British participants may limit generalizability to other populations.

## 5. Conclusions

In this large prospective cohort, higher social isolation, but not loneliness, was independently associated with increased AAA risk, with the highest absolute and relative risk observed among individuals who also had high polygenic susceptibility. Systemic immune-inflammation captured by SIRI modestly mediated this association, suggesting that structural social disconnection is a modifiable psychosocial factor that could be incorporated, alongside genetic risk, into AAA risk stratification and prevention.

## Figures and Tables

**Table 1:** Baseline characteristics of participants according to categories of the social isolation index.

Characteristics	Social Isolation Index			*p*-Value
	0	1	≥2	
*n*	196,790	131,874	27,517	
**Demographics**				
Age (years)	56.6 ± 8.05	56.4 ± 8.06	56.7 ± 7.74	<0.001
Male (%)	91,181 (46.3%)	60,030 (45.5%)	13,491 (49.0%)	<0.001
Townsend deprivation index	−2.00 ± 2.64	−1.35 ± 3.00	−0.43 ± 3.34	<0.001
University or college degree (%)	71,761 (36.5%)	43,951 (33.3%)	9149 (33.2%)	<0.001
Employed, student, or retired (%)	181,925 (92.4%)	120,696 (91.5%)	24,490 (89.0%)	<0.001
BMI	27.1 ± 4.32	27.4 ± 4.68	27.6 ± 5.03	<0.001
**Lifestyle**				
Healthy diet (%)	17,666 (8.9%)	13,146 (9.9%)	3150 (11.4%)	<0.001
No heavy alcohol (%)	81,802 (41.6%)	67,244 (51.0%)	16,528 (60.1%)	<0.001
Smoking status (%)				<0.001
Never	79,532 (40.4%)	51,892 (39.3%)	10,117 (36.8%)	
Previous	102,967 (52.3%)	66,325 (50.3%)	13,103 (47.6%)	
Current	14,291 (7.26%)	13,657 (10.4%)	4297 (15.6%)	
Physical activity (%)				<0.001
Low	51,083 (26.0%)	48,590 (36.8%)	12,260 (44.6%)	
Moderate	67,906 (34.5%)	43,825 (33.2%)	8539 (31.0%)	
High	77,801 (39.5%)	39,459 (29.9%)	6718 (24.4%)	
Sleep patterns (%)				<0.001
Poor	8694 (4.4%)	7154 (5.4%)	1974 (7.2%)	
Moderate	117,697 (59.8%)	80,797 (61.3%)	17,304 (62.9%)	
Good	70,399 (35.8%)	43,923 (33.3%)	8239 (29.9%)	
Sedentary time (%)				<0.001
High	36,163 (18.4%)	27,487 (20.8%)	7025 (25.5%)	
Moderate	65,713 (33.4%)	44,941 (34.1%)	9070 (33.0%)	
Low	94,914 (48.2%)	59,446 (45.1%)	11,422 (41.5%)	
**Medical history**				
Hypertension (%)	55,902 (28.4%)	40,049 (30.4%)	9024 (32.8%)	<0.001
Diabetes (%)	11,634 (5.9%)	9702 (7.4%)	2675 (9.7%)	<0.001
Dyslipidemia (%)	73,617 (37.4%)	51,450 (39.0%)	11,303 (41.1%)	<0.001
Psychiatric diseases (%)	14,480 (7.4%)	12,054 (9.1%)	3399 (12.4%)	<0.001
Chronic respiratory diseases (%)	24,836 (12.6%)	17,414 (13.2%)	3980 (14.5%)	<0.001
Chronic liver disease (%)	501 (0.3%)	444 (0.3%)	143 (0.5%)	<0.001
Chronic kidney disease (%)	524 (0.3%)	401 (0.3%)	118 (0.4%)	<0.001
Cardiovascular disease (%)	19,042 (9.7%)	13,500 (10.2%)	3223 (11.7%)	<0.001
Number of medications (SD)	2.2 ± 2.4	2.4 ± 2.5	2.6 ± 2.7	<0.001
Lipid-lowering medication (%)	28,012 (14.2%)	19,601 (14.9%)	4582 (16.7%)	<0.001
Antihypertensive medication (%)	37,928 (19.3%)	27,028 (20.5%)	6104 (22.2%)	<0.001
Antidiabetic medication (%)	4822 (2.45%)	4211 (3.19%)	1222 (4.44%)	<0.001
**Inflammation**				
Neutrophil count (10^9^/L)	4.1 ± 1.2	4.2 ± 1.2	4.4 ± 1.3	<0.001
Monocyte count (10^9^/L)	0.5 ± 0.1	0.5 ± 0.2	0.5 ± 0.2	<0.001
Lymphocyte count (10^9^/L)	1.9 ± 0.5	1.9 ± 0.5	1.9 ± 0.6	<0.001
Platelet count (10^9^/L)	251 ± 52.0	253 ± 53.0	254 ± 53.8	<0.001
SIRI	1.0 ± 0.6	1.1 ± 0.6	1.2 ± 0.6	<0.001
SII	570 ± 255	592 ± 267	611 ± 280	<0.001

*p* values were determined using the ANOVA test for continuous variables and the chi-square test for categorical variables. *p* values were used to describe overall differences across social isolation categories and were not intended for formal pairwise inference. Abbreviations: BMI, Body mass index; SIRI, Systemic Inflammation Response Index; SII, Systemic Immune-inflammation Index.

## Data Availability

This study was conducted under UK Biobank application number 145937. The UK Biobank data are available on application to the UK Biobank (www.ukbiobank.ac.uk/). The analytical code used in this study is available from the corresponding author upon reasonable request, in accordance with the UK Biobank data access policy.

## Supplementary Materials

The following supporting information can be downloaded at: https://www.mdpi.com/article/10.3390/healthcare14142126/s1, Supplementary Table S1: Lifestyle assessment, Table S2: Components of an ideal diet, Table S3: Drug classification and data coding, Table S4: Baseline characteristics of participants according to categories of the loneliness index, Table S5: Comparison of the predictive performance of social isolation and loneliness across different Cox models, Table S6: Subgroup associations of social isolation and loneliness with the risk of incident AAA, Table S7: Sensitivity analyses for the associations of social isolation and loneliness with AAA risk, Table S8: Association between PRS and risk of AAA, Table S9: Association between inflammatory index and the risk of AAA; Figure S1: Flowchart of study participants, Figure S2: Kaplan–Meier curves for cumulative incidence of AAA according to social isolation and loneliness indices, Figure S3: Distribution density plot of PRS.

## Abbreviations

The following abbreviations are used in this manuscript:

AAA	Abdominal aortic aneurysm
ARI	Absolute risk increase
BMI	Body mass index
CI	Confidence interval
GWAS	Genome-wide association study
HPA	Hypothalamic–pituitary–adrenal
HR	Hazard ratio
ICD-10	International Classification of Diseases, Tenth Revision
IL-6	Interleukin 6
NF-κB	Nuclear factor kappa B
PRS	Polygenic risk score
PRS-CS	Polygenic risk score continuous shrinkage
RERI	Relative excess risk due to interaction
SD	Standard deviation
SII	Systemic immune inflammation index
SIRI	Systemic inflammation response index
TDI	Townsend Deprivation Index
TNF-α	Tumor necrosis factor alpha

## References

[1] Kumar Y., Hooda K., Li S., Goyal P., Gupta N., Adeb M. (2017). Abdominal aortic aneurysm: Pictorial review of common appearances and complications. Ann. Transl. Med..

[2] Isselbacher E.M., Preventza O., Hamilton Black J., Augoustides J.G., Beck A.W., Bolen M.A., Braverman A.C., Bray B.E., Brown-Zimmerman M.M., Chen E.P. (2022). 2022 ACC/AHA Guideline for the Diagnosis and Management of Aortic Disease: A Report of the American Heart Association/American College of Cardiology Joint Committee on Clinical Practice Guidelines. Circulation.

[3] Mazzolai L., Teixido-Tura G., Lanzi S., Boc V., Bossone E., Brodmann M., Bura-Rivière A., De Backer J., Deglise S., Della Corte A. (2024). 2024 ESC Guidelines for the management of peripheral arterial and aortic diseases. Eur. Heart J..

[4] Anagnostakos J., Lal B.K. (2021). Abdominal aortic aneurysms. Prog. Cardiovasc. Dis..

[5] Holt-Lunstad J., Smith T.B. (2016). Loneliness and social isolation as risk factors for CVD: Implications for evidence-based patient care and scientific inquiry. Heart.

[6] Xia N., Li H. (2018). Loneliness, Social Isolation, and Cardiovascular Health. Antioxid. Redox Signal..

[7] Stepien K.L., Bajdak-Rusinek K., Fus-Kujawa A., Kuczmik W., Gawron K. (2022). Role of Extracellular Matrix and Inflammation in Abdominal Aortic Aneurysm. Int. J. Mol. Sci..

[8] Atkinson G., Bianco R., Di Gregoli K., Johnson J.L. (2023). The contribution of matrix metalloproteinases and their inhibitors to the development, progression, and rupture of abdominal aortic aneurysms. Front. Cardiovasc. Med..

[9] Shen C., Zhang R., Yu J., Sahakian B.J., Cheng W., Feng J. (2025). Plasma proteomic signatures of social isolation and loneliness associated with morbidity and mortality. Nat. Hum. Behav..

[10] Harrison S.C., Holmes M.V., Agu O., Humphries S.E. (2011). Genome wide association studies of abdominal aortic aneurysms-biological insights and potential translation applications. Atherosclerosis.

[11] Roychowdhury T., Klarin D., Levin M.G., Spin J.M., Rhee Y.H., Deng A., Headley C.A., Tsao N.L., Gellatly C., Zuber V. (2023). Genome-wide association meta-analysis identifies risk loci for abdominal aortic aneurysm and highlights PCSK9 as a therapeutic target. Nat. Genet..

[12] Raffort J., Lareyre F., Clément M., Hassen-Khodja R., Chinetti G., Mallat Z. (2017). Monocytes and macrophages in abdominal aortic aneurysm. Nat. Rev. Cardiol..

[13] Qin P., Ho F.K., Celis-Morales C.A., Pell J.P. (2025). Association between systemic inflammation biomarkers and incident cardiovascular disease in 423,701 individuals: Evidence from the UK biobank cohort. Cardiovasc. Diabetol..

[14] Cox N. (2018). UK Biobank shares the promise of big data. Nature.

[15] Zhou J., Hu X., Zhou S., Liu T., Chen Z. (2026). Social isolation, loneliness, genetic susceptibility, and the hazard of incident osteoporosis. Int. J. Surg..

[16] Allegrini A.G., Baldwin J.R., Barkhuizen W., Pingault J.B. (2022). Research Review: A guide to computing and implementing polygenic scores in developmental research. J. Child Psychol. Psychiatry.

[17] Elliott P., Peakman T.C. (2008). The UK Biobank sample handling and storage protocol for the collection, processing and archiving of human blood and urine. Int. J. Epidemiol..

[18] Qi Q., Zhuang L., Shen Y., Geng Y., Yu S., Chen H., Liu L., Meng Z., Wang P., Chen Z. (2016). A novel systemic inflammation response index (SIRI) for predicting the survival of patients with pancreatic cancer after chemotherapy. Cancer.

[19] Kong F., Huang J., Xu C., Huang T., Wen G., Cheng W. (2023). System inflammation response index: A novel inflammatory indicator to predict all-cause and cardiovascular disease mortality in the obese population. Diabetol. Metab. Syndr..

[20] Hu B., Yang X.R., Xu Y., Sun Y.F., Sun C., Guo W., Zhang X., Wang W.M., Qiu S.J., Zhou J. (2014). Systemic immune-inflammation index predicts prognosis of patients after curative resection for hepatocellular carcinoma. Clin. Cancer Res..

[21] Valtorta N.K., Kanaan M., Gilbody S., Ronzi S., Hanratty B. (2016). Loneliness and social isolation as risk factors for coronary heart disease and stroke: Systematic review and meta-analysis of longitudinal observational studies. Heart.

[22] Holt-Lunstad J., Smith T.B., Layton J.B. (2010). Social relationships and mortality risk: A meta-analytic review. PLoS Med..

[23] Steptoe A., Shankar A., Demakakos P., Wardle J. (2013). Social isolation, loneliness, and all-cause mortality in older men and women. Proc. Natl. Acad. Sci. USA.

[24] Cené C.W., Loehr L., Lin F.C., Hammond W.P., Foraker R.E., Rose K., Mosley T., Corbie-Smith G. (2012). Social isolation, vital exhaustion, and incident heart failure: Findings from the Atherosclerosis Risk in Communities Study. Eur. J. Heart Fail..

[25] Hawkley L.C., Cacioppo J.T. (2010). Loneliness matters: A theoretical and empirical review of consequences and mechanisms. Ann. Behav. Med..

[26] Kobayashi L.C., Steptoe A. (2018). Social Isolation, Loneliness, and Health Behaviors at Older Ages: Longitudinal Cohort Study. Ann. Behav. Med..

[27] Belsky D.W., Israel S. (2014). Integrating genetics and social science: Genetic risk scores. Biodemography Soc. Biol..

[28] Luo Y., Yang L., Cheng X., Bai Y., Xiao Z. (2025). The association between blood count based inflammatory markers and the risk of atrial fibrillation heart failure and cardiovascular mortality. Sci. Rep..

[29] Golledge J. (2019). Abdominal aortic aneurysm: Update on pathogenesis and medical treatments. Nat. Rev. Cardiol..

[30] Cole S.W. (2019). The Conserved Transcriptional Response to Adversity. Curr. Opin. Behav. Sci..

[31] Heck A.L., Sheng J.A., Miller A.M., Stover S.A., Bales N.J., Tan S.M.L., Daniels R.M., Fleury T.K., Handa R.J. (2020). Social isolation alters hypothalamic pituitary adrenal axis activity after chronic variable stress in male C57BL/6 mice. Stress.

[32] Sher L.D., Geddie H., Olivier L., Cairns M., Truter N., Beselaar L., Essop M.F. (2020). Chronic stress and endothelial dysfunction: Mechanisms, experimental challenges, and the way ahead. Am. J. Physiol.-Heart Circ. Physiol..

[33] Moieni M., Irwin M.R., Jevtic I., Breen E.C., Cho H.J., Arevalo J.M., Ma J., Cole S.W., Eisenberger N.I. (2015). Trait sensitivity to social disconnection enhances pro-inflammatory responses to a randomized controlled trial of endotoxin. Psychoneuroendocrinology.

