# Supplementary material for: Social Isolation, Genetic Susceptibility, Systemic Inflammation and Risk of Abdominal Aortic Aneurysm: A UK Biobank Cohort Study
Source: Healthcare (Basel). 2026 Jul 15;14(14):2126. doi: 10.3390/healthcare14142126 (PMC13409830; doi:10.3390/healthcare14142126)

Supplementary Material

Supplementary Table S1. Lifestyle assessment

Supplementary Table S2. Components of an ideal diet

Supplementary Table S3. Drug classification and data coding

Supplementary Table S4. Baseline characteristics of participants according to categories of the loneliness index

Supplementary Table S5. Comparison of the predictive performance of social isolation and loneliness across different Cox models

Supplementary Table S6. Subgroup associations of social isolation and loneliness with the risk of incident AAA

Supplementary Table S7. Sensitivity analyses for the associations of social isolation and loneliness with AAA risk

Supplementary Table S8. Association between PRS and risk of AAA

Supplementary Table S9. Association between inflammatory index and the risk of AAA

Supplementary Figure S1. Flowchart of study participants

Supplementary Figure S2. Kaplan–Meier curves for cumulative incidence of AAA according to social isolation and loneliness indices

Supplementary Figure S3. Distribution density plot of PRS

Supplementary Table S1. Lifestyle assessment

**Lifestyle factor**	**Field IDs**	**Lifestyle assessment**
Smoking	1239<br>1249<br>2644	Categorized as never smokers, former smokers, and current smokers.
Drinking	1558<br>1568<br>1578<br>1588<br>1598<br>1608<br>5364	More than two drinks per day for men<br>More than one drink per day for women
Sleep pattern	1160<br>1180<br>1200<br>1210<br>1220	For each component of the sleep pattern, a score of 1 is assigned to high-risk components, while low-risk components are assigned a score of 0. The total sleep pattern score ranges from 0 to 5, with scores of 0–1 indicating a low-risk category, 2–3 representing a medium-risk category, and 4–5 corresponding to high-risk sleep patterns.
Physical activity	971<br>981<br>991<br>1001<br>2624<br>2634<br>3637<br>3647	The cohort was categorized into light, moderate, and heavy physical activity levels based on the tertile distribution of weekly physical activity Metabolic Equivalent of Task (MET) values.
Sedentary time	1070<br>1080	The cohort was divided into mild, moderate, and severe levels of sedentary behavior based on the tertile distribution of sedentary time.
Diet	Supplementary table S2	A diet consisting of five or more key components is considered a healthy diet.

Supplementary Table S2. Components of an ideal diet

Diet component	Field IDs	Intake goal
Fruit	1309<br>1319	≥3 servings/day
Vegetable	1289<br>1299	≥3 servings/day
Whole grains	1438/1448<br>1458/1468	≥3 servings/day
Fish (Shell)	1329<br>1339	≥2 servings/day
Dairy	1408<br>1418	≥2 servings/day
Vegetable oils	1428<br>1438<br>2654	≥2 servings/day
Refined grains	1438/1448<br>1458/1468	≤2 servings/day
processed meats	1349<br>3680	≤1 servings/day
Unprocessed meats	1359<br>1369<br>1379<br>1389<br>3680	≤2 servings/day
Sugar-sweetened beverages	6144	No consumption

Supplementary Table S3. Drug classification and data coding

Drug Classification	Data Code
Lipid-lowering medication	20003, 6177, 6153 (1140861922; 1141146234; 1140910654; 1141192410; 1140861958; 1140888594; 1140888648; 1140861970; 1140910632; 140861946;1140862028; 1141192414; 1140861924; 1140861954; 1141157260; 1140861944; 1140861856; 1141157262; 1140861926; 1140861928; 1140861936; 1140861942; 140861858;1140888590;1140910652;1140861868;1140861892;1140861876; 1140861866; 1140861848; 1140861878;1140861894; 1140865576;1140909780; 1141157416)
Antihypertensive medication	20003, 6177, 6153 (1140851692; 1140860696; 1140860706; 1140860714; 1140860728; 1140860736; 1140860750; 1140860752; 1140860758; 1140860764; 1140860776; 1140860784; 1140860790; 1140860802; 1140860806; 1140860878; 1140860882; 1140860892; 1140860904; 1140860912; 1140860918; 1140864618; 1140864910; 1140864952; 1140866078; 1140879802; 1140881706; 1140888552; 1140888556; 1140888560; 1140888646; 1140916356; 1140916362; 1140923718; 1141145660; 1141145668; 1141150328; 1141150560; 1141151016; 1141151018; 1141152998; 1141153006; 1141153316; 1141153328; 1141156836; 1141156846; 1141164148; 1141164154; 1141165470; 1141165476; 1141166006; 1141167758; 1141167822; 1141170544; 1141170870; 1141171336; 1141172492; 1141172682; 1141172686; 1141179974; 1141180592; 1141180598; 1141187788; 1141188408; 1141190934; 1141193282; 1141193346; 1141199940; 1141200698; 1141200726; 1141201038; 1141201040; 1140851332; 1140851336; 1140851338; 1140851362; 1140851364; 1140851368; 1140851660; 1140860332; 1140860404; 1140860422; 1140860562; 1140860738; 1140864950; 1140866072; 1140866074; 140866084; 1140866086; 1140866092; 1140866096; 1140866108; 1140866110; 1140866128; 1140866132; 1140866136; 1140866138; 1140866140; 1140866146; 1140866156; 1140866158; 1140866162; 1140866164; 1140866168; 1140866422; 1140866440; 1140866446; 1140888918; 1140888922; 1140909706; 1140916870; 1140917068; 1140926778; 1141146378; 1141180772; 1141180778; 1141188636; 1141194794; 1141194800; 1141194804; 1141194808; 1141194810; 1140851360; 1140851418; 1140851420; 1140851428; 1140851430; 1140851436; 1140860292; 1140860322; 1140860336;1140864550; 1140864574; 1140866220; 1140866226; 1140866232; 1140866236; 1140866244; 1140866248; 1140866280; 1140866282; 1140866306; 1140866308; 1140866312; 1140866318; 1140866324; 1140866328; 1140866330; 1140866332; 1140866334; 1140866340; 1140866352; 1140866354; 1140866356; 1140866360; 1140866388; 1140866390; 1140866396; 1140866400; 1140866402; 1140866404; 1140866406; 1140866410; 1140866416; 1140866418; 1140866420; 1140866426; 1140866438; 1140866442; 1140866444; 1140866448; 1140881894; 1140888512; 1140909708; 1140909722; 1140922324; 1140923272; 1140923276; 1140923282; 1140923402; 1140927174; 1140928624; 1141146128; 1141150898; 1141167108; 1141181520; 1141195254; 1141195258; 1141201250; 1140851508; 1140851522; 1140851556; 1140860172; 1140860180; 1140860232; 1140860250; 1140860266; 1140860278; 1140860308; 1140860314; 1140860318; 1140860320; 1140860324; 1140860328; 1140860330; 1140860338; 1140860340; 1140860342; 1140860348; 1140860356; 1140860358; 1140860386; 1140860394; 1140860396; 1140860398; 1140860402; 1140860406; 1140860410; 1140860426; 1140860434; 1140860492; 1140860498; 1140861090; 1140864176; 1140864410; 1140866704; 1140866712; 1140866724; 1140866738; 1140866756; 1140866758; 1140866764; 1140866766; 1140866778; 1140866782; 1140866784; 1140866798;

	1140866800; 1140866802; 1140866804; 1140867734; 1140879758; 1140879760; 1140879762; 1140879818; 1140879824; 1140879834; 1140879854; 1140879866; 1140881702; 1140881722; 1140909368; 1140916628; 1140916730; 1140916868; 1140917076; 1140922930; 1140923336; 1141146124; 1141146126; 1141146184; 1141152076; 1141156754; 1141164280; 1141168498; 1141169516; 1141171152; 1141172742; 1141182904; 1141182968; 1141184324; 1141184722; 1141187048; 1141187780; 1140851730; 1140851784; 1140851786; 1140851790; 1140851800; 1140855976; 1140861088; 1140861106; 1140861110; 1140861114; 1140861120; 1140861128; 1140861130; 1140861136; 1140861138; 1140861176; 1140861190; 1140861202; 1140861276; 1140861282; 1140866460; 1140866466; 1140866484; 1140866546; 1140866554; 1140868036; 1140872472; 1140872568; 1140879810; 1140881692; 1140888510; 1140911088; 1140911698; 1140916930; 1140917428; 1140923572; 1140923618; 1140926188; 1140926780; 1140927934; 1140927940; 1140928212; 1140928226; 1140928234; 1141145870; 1141150500; 1141150538; 1141150926; 1141151474; 1141152600; 1141153026; 1141153032; 1141153394; 1141153454; 1141156656; 1141157136; 1141157140; 1141162546; 1141166752; 1141167832; 1141169096; 1141169710; 1141169730; 1141171804; 1141173766; 1141174684; 1141175224; 1141180238; 1141184390; 1141185444; 1141187056; 1141187094; 1141187774; 1141187962; 1141188152; 1141188576; 1141188936; 1141190548; 1141199858; 1141200400; 1141200782; 1141201814)
Antidiabetic medication	20003, 6177, 6153 (1140857494; 1140874724; 1140857496; 1140874726;1140857500;1140874728; 1140857502; 1140874732; 1140857506; 1140874736; 1140874646; 1140874744; 1140874650; 1140874746; 1140874652; 1140883066; 1140874664; 1140884600; 1140874666; 1141152590; 1140874674; 1141153254; 1140874678; 1141153262; 1140874680; 1141156984; 1140874686; 1141171646; 1140874690; 1141171652; 1140874706; 1141177600; 1140874712; 1141177606; 1140874716; 1141189090; 1140874718; 1141189094)

Supplementary Table S4. Baseline characteristics of participants according to categories of the loneliness index

Characteristics	Loneliness index			*P*-value
	0	1	2	
N	248607	87957	19617	
**Demographics**				
Age (years)	56.5±8.04	56.8±8.02	56.0±7.91	< 0.001
Male (%)	113449 (45.6%)	41758 (47.5%)	9495 (48.4%)	< 0.001
Townsend deprivation index	-1.79±2.77	-1.37±3.02	-0.90±3.23	< 0.001
University or college degree (%)	92614 (37.3%)	26923 (30.6%)	5324 (27.1%)	< 0.001
Employed, student, or retired (%)	230876 (92.9%)	79459 (90.3%)	16776 (85.5%)	< 0.001
BMI	27.1±4.40	27.5±4.69	28.0±5.03	< 0.001
**Lifestyle**				
Healthy diet (%)	23529 (9.5%)	8544 (9.7%)	1889 (9.6%)	0.086
No heavy alcohol (%)	111344 (44.8%)	43665 (49.6%)	10565 (53.9%)	< 0.001
Smoking status (%)				< 0.001
Never	100198 (40.3%)	34126 (38.8%)	7217 (36.8%)	
Previous	128495 (51.7%)	44318 (50.4%)	9582 (48.8%)	
Current	19914 (8.01%)	9513 (10.8%)	2818 (14.4%)	
Physical activity (%)				< 0.001
Low	74028 (29.8%)	30340 (34.5%)	7565 (38.6%)	
Moderate	84937 (34.2%)	29012 (33.0%)	6321 (32.2%)	
High	89642 (36.1%)	28605 (32.5%)	5731 (29.2%)	
Sleep patterns (%)				< 0.001
Poor	10172 (4.1%)	5696 (6.5%)	1954 (9.9%)	
Moderate	146317 (58.9%)	56258 (64.0%)	13223 (67.4%)	
Good	92118 (37.1%)	26003 (29.6%)	4440 (22.6%)	
Sedentary time (%)				< 0.001
High	45789 (18.4%)	19744 (22.4%)	5142 (26.2%)	

Moderate	82765 (33.3%)	30255 (34.4%)	6704 (34.2%)	
Low	120053 (48.3%)	37958 (43.2%)	7771 (39.6%)	
**Medical history**				
Hypertension (%)	71015 (28.6%)	27297 (31.0%)	6663 (34.0%)	< 0.001
Diabetes (%)	15475 (6.2%)	6715 (7.6%)	1821 (9.3%)	< 0.001
Dyslipidemia (%)	92831 (37.3%)	35177 (40.0%)	8362 (42.6%)	< 0.001
Psychiatric diseases (%)	16254 (6.54%)	10333 (11.7%)	3346 (17.1%)	< 0.001
Chronic respiratory diseases (%)	31147 (12.5%)	11992 (13.6%)	3091 (15.8%)	< 0.001
Chronic liver disease (%)	668 (0.2%)	327 (0.3%)	93 (0.4%)	< 0.001
Chronic kidney disease (%)	681 (0.2%)	273 (0.3%)	89 (0.4%)	< 0.001
Cardiovascular disease (%)	23311 (9.4%)	9853 (11.2%)	2601 (13.3%)	< 0.001
Number of medications (SD)	2.2±2.4	2.5±2.6	2.8±2.8	< 0.001
Lipid-lowering medication (%)	35098 (14.1%)	13798 (15.7%)	3299 (16.8%)	< 0.001
Antihypertensive medication (%)	48217 (19.4%)	18463 (21.0%)	4380 (22.3%)	< 0.001
Antidiabetic medication (%)	6460 (2.60%)	2916 (3.32%)	879 (4.48%)	< 0.001
**Inflammation**				
Neutrophil count (10^9/L)	4.1±1.2	4.2±1.2	4.3±1.3	< 0.001
Monocyte count (10^9/L)	0.5±0.1	0.5±0.2	0.5±0.2	< 0.001
Lymphocyte count (10^9/L)	1.9±0.5	1.9±0.5	1.9±0.6	< 0.001
Platelet count (10^9/L)	251±52.4	253±52.8	254±53.4	< 0.001
SIRI	1.0±0.6	1.1±0.6	1.1±0.6	< 0.001
SII	577±260	591±265	601±271	< 0.001

P values were determined using the ANOVA test for continuous variables and the chi-square test for categorical variables. P values were used to describe overall differences across social isolation categories and were not intended for formal pairwise inference. Abbreviations: BMI, Body mass index; SIRI, Systemic Inflammation Response Index; SII, Systemic Immune-inflammation Index.

Supplementary Table S5. Comparison of the predictive performance of social isolation and loneliness across different Cox models

Model	Social isolation index		Loneliness index	
	**Categorical variable with three levels**	**Per SD increment**	**Categorical variable with three levels**	**Per SD increment**
**Model 1**				
Schoenfeld Test (P-value)	0.151	0.122	0.112	0.106
C-index	0.837	0.837	0.836	0.836
BIC	41233.68	41222.57	41251.55	41244.18
**Model 2**				
Schoenfeld Test (P-value)	0.423	0.313	0.172	0.165
C-index	0.861	0.861	0.861	0.861
BIC	40732.28	40722.51	40748.49	40741.52
**Model 3**				
Schoenfeld Test (P-value)	0.155	0.221	0.241	0.212
C-index	0.882	0.882	0.881	0.881
BIC	40057.17	40048.27	40061.31	40055.02

Model 1: Adjusted for sex and age. Model 2: Further adjusted for education level, employment status, Townsend deprivation index, body mass index, medication history, and medical history (including hypertension, diabetes, dyslipidemia, cardiovascular diseases, psychiatric diseases, chronic respiratory diseases, chronic kidney disease, and chronic liver disease). Model 3: Additionally adjusted for smoking status, alcohol consumption, physical activity, diet, sleep patterns, and sedentary time. Abbreviations: BIC, Bayesian Information Criterion.

Supplementary Table S6. Subgroup associations of social isolation and loneliness with the risk of incident AAA

Subgroup	Incident AAA#	Social isolation index			Loneliness index		
		Index =/≥ 2 vs Index = 0	Per SD increment	$P_{interaction}$	Index =/≥ 2 vs Index = 0	Per SD increment	$P_{interaction}$
**Sex**				0.287			0.098
Male	0.577	1.22 (1.02-1.46)	1.10 (1.02-1.19)		1.01 (0.81-1.24)	1.03 (0.95-1.13)	
Female	0.088	1.24 (0.82-1.85)	1.13 (0.94-1.35)		1.35 (0.87-2.09)	1.17 (0.97-1.41)	
**Age**				0.137			0.431
≥ 60	0.607	1.14 (0.95-1.38)	1.07 (0.99-1.17)		1.01 (0.79-1.25)	1.05 (0.97-1.15)	
< 59	0.096	1.41 (1.01-2.02)	1.19 (1.01-1.39)		1.01 (0.68-1.50)	1.01 (0.85-1.19)	
**BMI**				0.303			0.437
Normal BMI	0.158	1.28 (0.82-1.99)	1.19 (0.97-1.44)		1.08 (0.63-1.85)	1.14 (0.92-1.41)	
Abnormal BMI	0.358	1.23 (1.03-1.46)	1.10 (1.02-1.19)		1.05 (0.85-1.29)	1.05 (0.96-1.34)	
**Hypertension**				0.852			0.433
No	0.193	1.24 (1.01-1.61)	1.14 (1.02-1.26)		1.13 (0.84-1.53)	1.07 (0.95-1.21)	
Yes	0.601	1.24 (1.03-1.54)	1.10 (1.01-1.21)		1.02 (0.79-1.31)	1.06 (0.95-1.17)	
**Smoking status**				0.524			0.822
Never	0.115	1.38 (1.03-1.61)	1.22 (1.01-1.46)		1.01 (0.56-1.77)	1.08 (0.88-1.33)	

Previous	0.344	1.24 (1.01-1.57)	1.11 (1.01-1.22)	0.98 (0.74-1.31)	1.06 (0.95-1.18)
Current	1.016	1.09 (0.83-1.44)	1.05 (0.93-1.20)	1.13 (0.83-1.53)	1.04 (0.91-1.19)

[#]Incidence rates per 1,000 person-years. All analyses were adjusted for sex, age, education level, employment status, Townsend deprivation index, body mass index, medication history, medical history (including hypertension, diabetes, dyslipidemia, cardiovascular diseases, psychiatric diseases, chronic respiratory diseases, chronic kidney disease, and chronic liver disease), as well as smoking status, alcohol consumption, physical activity, diet, sleep patterns, and sedentary time. The corresponding covariates were excluded in each relevant subgroup analysis.

Supplementary Table S7. Sensitivity analyses for the associations of social isolation and loneliness with AAA risk

Analyses	Social isolation index			Loneliness index		
	HR (95% CI)	*P*-value	Schoenfeld Test (P value)	HR (95% CI)	*P*-value	Schoenfeld Test (P value)
**Excluding participants with a prior medical history**						
Index =/≥ 2 vs Index = 0	1.57 (1.05-2.49)	0.037	0.597	0.85 (0.41-1.74)	0.657	0.359
Per SD increment	1.21 (1.02-1.47)	0.042	0.646	0.98 (0.76-1.26)	0.883	0.216
**Excluding events that occurred within the first two years of follow-up**						
Index =/≥ 2 vs Index = 0	1.17 (1.01-1.21)	0.041	0.102	1.02 (0.83-1.23)	0.835	0.604
Per SD increment	1.09 (1.01-1.17)	0.021	0.263	1.05 (0.97-1.14)	0.251	0.361
**Competing risk regression model**						
Index =/≥ 2 vs Index = 0	1.10 (1.02-1.18)	0.011	NA	1.06 (0.98-1.15)	0.121	NA
Per SD increment	1.11 (1.03-1.19)	0.005	NA	1.06 (0.98-1.14)	0.119	NA
**Multiple imputation**						
Index =/≥ 2 vs Index = 0	1.14 (1.05-1.23)	0.012	0.071	1.06 (0.87-1.28)	0.589	0.961
Per SD increment	1.12 (1.04-1.19)	0.002	0.095	1.06 (0.98-1.15)	0.143	0.782
**Additionally adjusted for antihypertensive, lipid-lowering, and antidiabetic medication use**						
Index =/≥ 2 vs Index = 0	1.24 (1.05-1.46)	0.010	0.067	1.07 (0.88-1.30)	0.476	0.716

Per SD increment	1.12 (1.04-1.20)	0.002	0.072	1.07 (0.99-1.15)	0.106	0.658

All analyses were adjusted for sex, age, education level, employment status, Townsend deprivation index, body mass index, medication history, medical history (including hypertension, diabetes, dyslipidemia, cardiovascular diseases, psychiatric diseases, chronic respiratory diseases, chronic kidney disease, and chronic liver disease), as well as smoking status, alcohol consumption, physical activity, diet, sleep patterns, and sedentary time.

Supplementary Table S8. Association between PRS and risk of AAA

Model	Tertile 1	Tertile 2	Tertile 3	P_{trend}	Schoenfeld Test (P-value)
**PRS-CS**					
Incident AAA[#]	0.145	0.229	0.561		
Model 1	Ref.	1.60 (1.37-1.86)	3.97 (3.47-4.54)	<0.001	0.121
Model 2	Ref.	1.56 (1.34-1.81)	3.77 (3.29-4.31)	<0.001	0.132
Model 3	Ref.	1.55 (1.33-1.81)	3.75 (3.28-4.29)	<0.001	0.145

[#]Incidence rates per 1,000 person-years. Model 1: Adjusted for sex, age, and the first ten genetic principal components. Model 2: Further adjusted for education level, employment status, Townsend deprivation index, body mass index, medication history, and medical history (including hypertension, diabetes, dyslipidemia, cardiovascular diseases, psychiatric diseases, chronic respiratory diseases, chronic kidney disease, and chronic liver disease). Model 3: Additionally adjusted for smoking status, alcohol consumption, physical activity, diet, sleep patterns, and sedentary time.

Supplementary Table S9. Association between inflammatory index and the risk of AAA

Inflammatory markers	Incident AAA#	Model 1	Model 2	Model 3
**SII**				
Quartile 1	0.304	Ref.	Ref.	Ref.
Quartile 2	0.383	1.01 (0.87-1.14)	0.97 (0.85-1.11)	0.95 (0.83-1.09)
Quartile 3	0.311	1.07 (0.93-1.22)	1.02 (0.89-1.17)	0.99 (0.86-1.13)
Quartile 4	0.336	1.11 (0.97-1.26)	1.01 (0.89-1.16)	0.98 (0.86-1.12)
P for trend		0.077	0.712	0.875
Schoenfeld Test (P-value)		0.201	0.229	0.217
**SIRI**				
Quartile 1	0.144	Ref.	Ref.	Ref.
Quartile 2	0.231	1.21 (1.01-1.44)	1.14 (0.95-1.36)	1.09 (0.92-1.30)
Quartile 3	0.339	1.43 (1.21-1.69)	1.28 (1.08-1.51)	1.20 (1.01-1.41)
Quartile 4	0.539	1.69 (1.44-1.98)	1.38 (1.18-1.62)	1.24 (1.06-1.46)
P for trend		<0.001	<0.001	<0.001
Schoenfeld Test (P-value)		0.103	0.125	0.217

#Incidence rates per 1,000 person-years. Model 1: Adjusted for sex and age. Model 2: Further adjusted for education level, employment status, Townsend deprivation index, body mass index, medication history, and medical history (including hypertension, diabetes, dyslipidemia, cardiovascular diseases, psychiatric diseases, chronic respiratory diseases, chronic kidney disease, and chronic liver disease). Model 3: Additionally adjusted for smoking status, alcohol consumption, physical activity, diet, sleep patterns, and sedentary time. Abbreviations: SII, Systemic Immune-Inflammation Index; SIRI, Systemic Inflammation Response Index.

Supplementary Figure S1. Flowchart of study participants

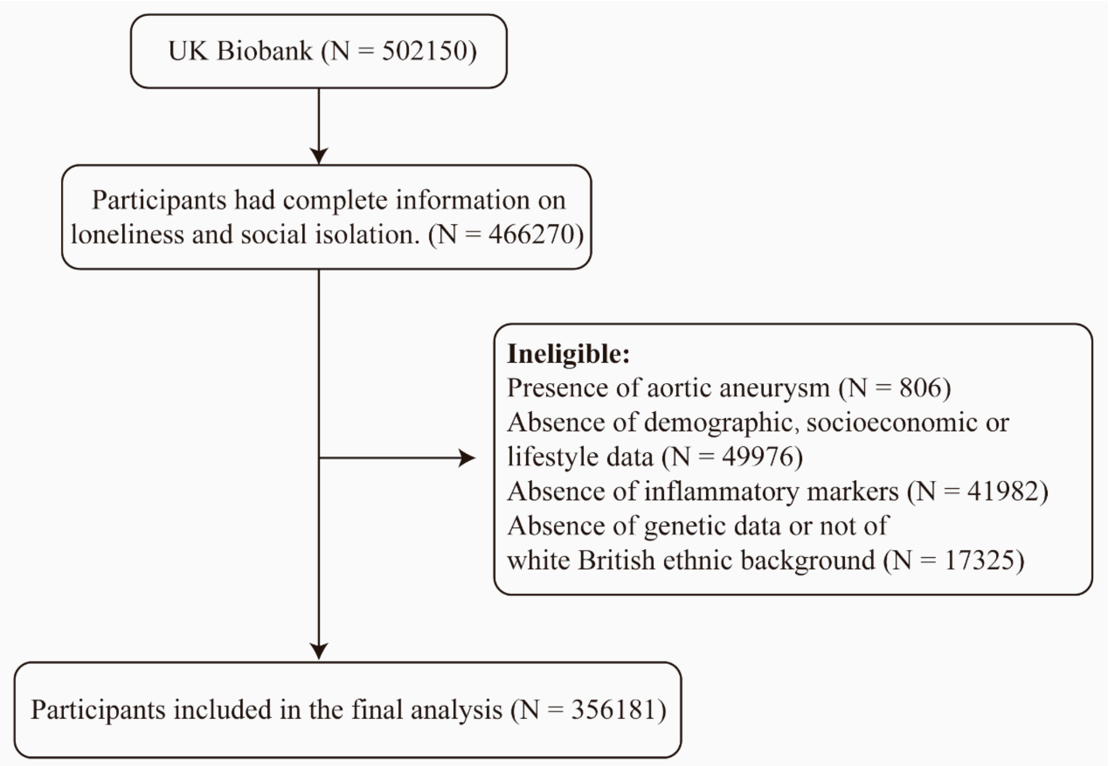

Supplementary Figure S2. Kaplan–Meier curves for cumulative incidence of AAA according to social isolation and loneliness indices

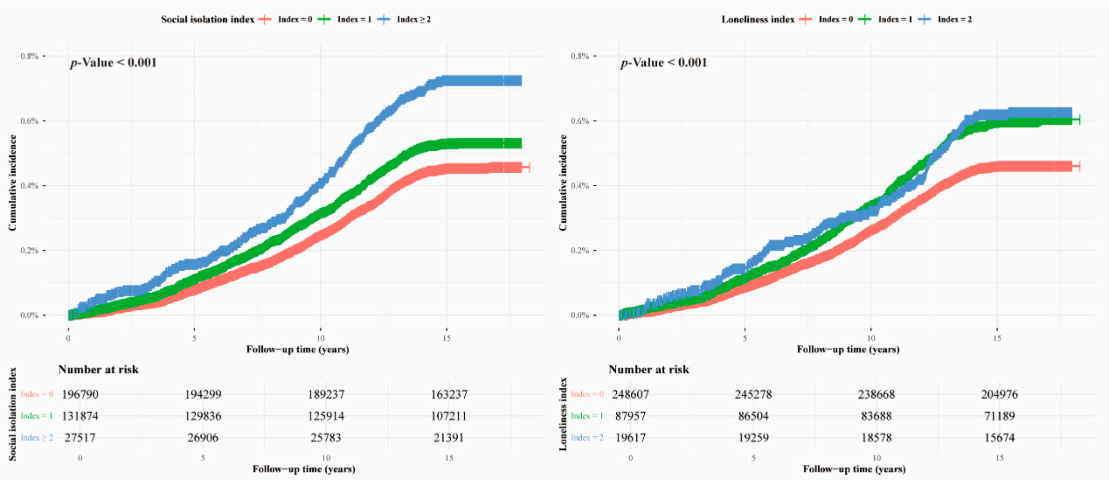

Supplementary Figure S3. Distribution density plot of PRS

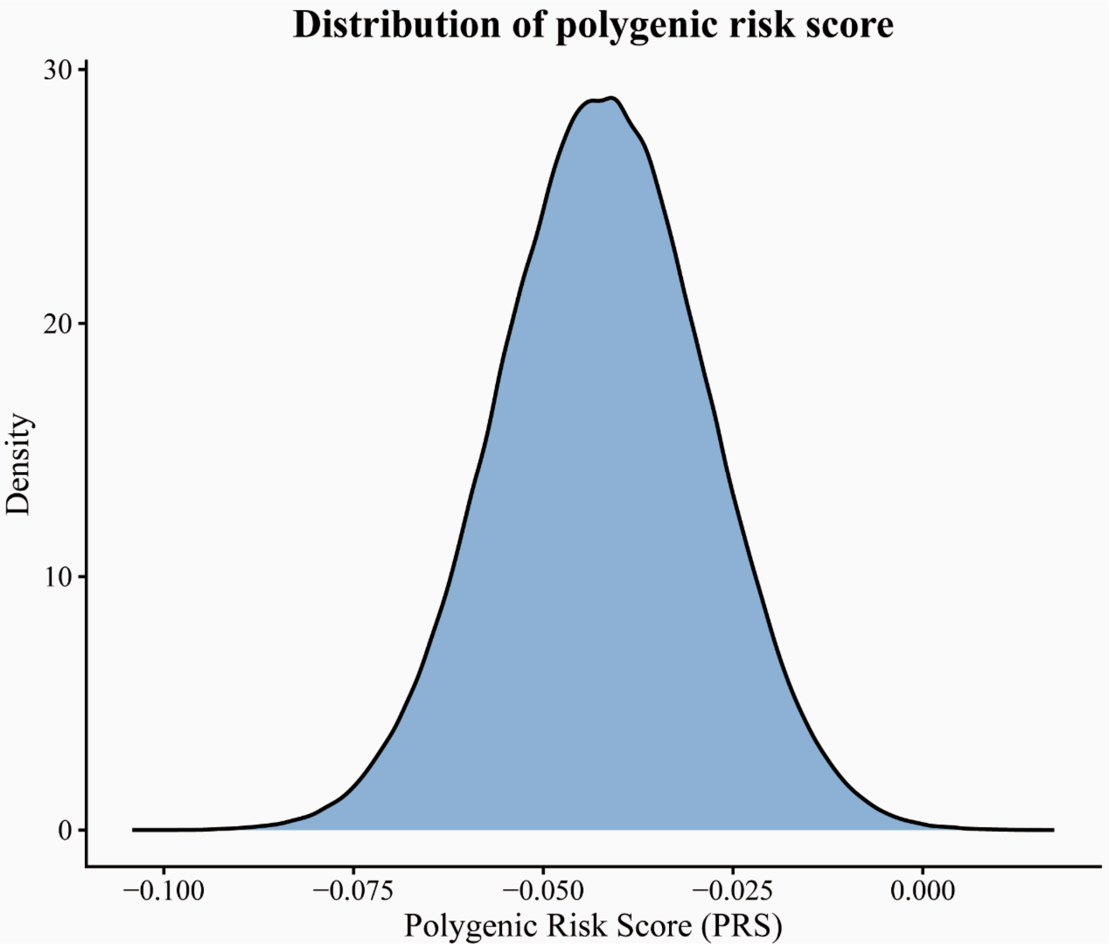

Supplement: Supplementary file 1 [file healthcare-14-02126-s001.zip › healthcare-4320082-supplementary.pdf]
